# A stamp of approval is not a seal of ethics: an editorial on justice and respect in trial design

**DOI:** 10.1186/s13063-025-09318-3

**Published:** 2025-11-24

**Authors:** Cory E. Goldstein, Shaun Treweek, Hossein Fatemian

**Affiliations:** 1 Methodological and Implementation Research Program, Ottawa Hospital Research Institute, Ottawa, Canada; 2 School of Epidemiology and Public Health, University of Ottawa, Ottawa, Canada; 3 Aberdeen Centre for Evaluation, University of Aberdeen, Aberdeen, UK; 4 Department of Women’s and Children’s Health, Uppsala University, Uppsala, Sweden; 5 Shiraz University of Medical Sciences, Shiraz, Iran

*Trials* receives hundreds of randomised trial protocols every year. Most do not reach the senior editorial team, but a recent protocol prompted a discussion about whether the journal should always accept local research ethics committee (REC) approval. The protocol described a randomised trial evaluating an antenatal educational program for adolescent (≤ 19 years), first-time mothers, with the aim of increasing exclusive breastfeeding and improving breastfeeding knowledge, attitudes, and practices.

Although the study had local REC approval, two features flagged during editorial assessment warranted closer scrutiny: eligibility was limited to married adolescents, and the investigators proposed to obtain consent not only from participants but also from their husbands on the basis that the participants are minors. These provisions engage ethical principles of justice and respect for persons—namely, whether marital status can ethically determine access to a health-education trial involving a vulnerable group, who constitutes an appropriate surrogate decision-maker for minors, and what role, if any, a spouse should play in authorising research participation. They also raise the broader question facing international journals of how to balance deference to local REC review with the obligation to apply coherent editorial standards across jurisdictions. In light of these concerns, the editorial team invited cross-disciplinary commentary to guide a transparent decision.

## Can a clinical trial be considered ethical in one jurisdiction but not another?

The short answer is no, and the reason lies in distinguishing ethics from regulation. RECs review protocols largely for conformity with local laws, policies, and customs; approval signals regulatory compliance, but not necessarily that a study attains high ethical standards.

Unsurprisingly, REC judgments can vary across institutions and countries, with the same protocol sometimes receiving different determinations [1–3]. By contrast, the ethical appraisal of research involving human participants turns on universal principles—respect for persons, beneficence, and justice—first articulated in the Belmont Report [4] and elaborated in other international ethics guidelines such as the Declaration of Helsinki [5] and the CIOMS International Ethical Guidelines [6]. These documents are intended to apply across settings and disciplines, providing a common ethical framework for assessing consent practices, benefit-harm analyses, and participant selection procedures.

Consequently, while regulatory outcomes may differ by jurisdiction, whether a trial is ethical should not: that determination rests on whether the study upholds these shared principles in design and conduct. We can therefore consider the submitted protocol through this lens and return to the two concrete questions raised during editorial assessment. We will then turn to the role of journal editors.

## Is it ethically permissible to exclude unmarried women from research?

In the protocol we received, the investigators did not offer a substantive justification for excluding unmarried adolescents. Even if motivated by prevailing laws or social stigma, exclusion on the basis of marital status lacks a scientific rationale. International ethics guidelines require all eligibility criteria to be grounded in science and not in potentially discriminatory categories [6]. It also recommends responsible inclusion of vulnerable participants over presumptive exclusion [6]. Moreover, justice demands that the benefits and burdens of research be distributed equitably. Unequal access—particularly when it applies to groups under-represented in research—must be explicitly justified, and the harms of exclusion must be weighed alongside any alleged harms of inclusion.

In an educational trial aimed at breastfeeding knowledge, attitudes, and practices, the risk–benefit profile of study participation does not plausibly hinge on marital status. Absent a study-specific, evidence-based rationale, barring unmarried, first pregnancy adolescents is ethically indefensible. Their inclusion, however, would pose social and legal risks in the country where the trial was being conducted. Therefore, protecting unmarried participants from social or legal risks should be addressed by robust privacy and confidentiality safeguards (e.g., minimizing or removing identifiers, limiting access to data, and clear disclosure policies). On balance, without a compelling and study-specific justification, excluding unmarried women from this trial is unethical.

## Should informed consent be obtained from spouses of adolescent women?

Respect for persons requires that capable individuals authorise their own participation; third-party “permission” is neither necessary nor ethically appropriate. International ethics guidance is explicit: “Only the informed consent of the woman herself should be required for her research participation… In no case must the permission of another person replace the requirement of individual informed consent” [6]. When adolescents are near the age of majority and can understand the study, their agreement is ethically equivalent to informed consent. (Where law requires a guardian, that role is ordinarily fulfilled by a parent or legally authorised representative, not a spouse, and adolescents’ own interests should be respected through assent procedures). Decision-making capacity, not age, determines who may provide informed consent. This means that individuals should be presumed capable unless shown otherwise by a task and context-specific capacity assessment. If a prospective participant truly lacks decisional capacity, informed consent should be sought from an appropriate legally authorised representative, and participant assent should be obtained whenever possible.

Accordingly, in trials involving adolescents—especially trials evaluating low-risk educational interventions—researchers should obtain the prospective participant’s informed consent when she is capable. If incapable of decision making, researchers should seek permission from an appropriate guardian or legally authorised representative and honour the adolescent’s assent or dissent. Spousal “permission” may never substitute for the participant’s informed consent, and requiring spousal consent alongside the participant’s consent risks coercion or undue influence that calls into question the voluntariness of the participant’s decision.

## What should journal editors do when they have concerns about the ethics of a trial?

Editors should separate regulatory compliance from ethical acceptability, and then act within clear, internationally accepted standards.

Concretely, this means that when a potential ethical issue is identified in a submitted protocol, editors should consider following COPE’s guidance and step-by-step flowcharts for suspected ethics issues [7]: pause peer review to request clarifications and documentary evidence (e.g., REC approvals, consent materials), and where concerns persist due to no or an unsatisfactory response, suspend peer review and consider forwarding the concern(s) to the authors’ institution.

Documenting the editorial decision and reasoning is also prudent. Leading editorial frameworks (e.g., ICMJE [8] and BMC’s [9] policies) expect journals to only publish research that meets contemporary ethical standards. As Higgins and colleagues have argued compellingly, “publishers have responsibilities to not publish contemporary unethical biomedical research, and where this has occurred, to retract publications” [10]. Journal editors bear a positive duty not to publish contemporary unethical research and to retract those that slip through because publication legitimises, disseminates, and potentially rewards ongoing unethical practice. That said, when a submission raises contestable ethical questions that can be constructively addressed (for example, through strengthened consent processes or fairer inclusion criteria), publication paired with an accompanying editorial or commentary can be appropriate to surface the issues and nudge practice toward higher ethical standards.

With regard to the protocol that prompted this editorial, *Trials* followed COPE guidance and requested additional information from the authors. Additional information was not provided, and *Trials* rejected the protocol.

## Data Availability

Not applicable.
